# Real-time PCR biochip for on-site detection of *Coxiella burnetii* in ticks

**DOI:** 10.1186/s13071-021-04744-z

**Published:** 2021-05-06

**Authors:** A.-Tai Truong, Bo-Ram Yun, Jiyeon Lim, Subin Min, Mi-Sun Yoo, Soon-Seek Yoon, Young-Min Yun, Jong-Taek Kim, Yun Sang Cho

**Affiliations:** 1 Parasitic and Honeybee Disease Laboratory, Bacterial and Parasitic Disease Division, Department of Animal & Plant Health Research, Animal and Plant Quarantine Agency, Gimcheon, 39660 Republic of Korea; 2 Department of Veterinary Internal Medicine, College of Veterinary Medicine, Jeju National University, Jeju, 63243 Republic of Korea; 3 College of Veterinary Medicine, Gangwon National University, Chuncheon, 24341 Republic of Korea; 4 Faculty of Biotechnology, Thai Nguyen University of Sciences, Thai Nguyen, 250000 Vietnam

**Keywords:** Q fever, Asian longhorned tick, *Haemaphysalis longicornis*, Tick-borne diseases, Ultra-rapid real-time PCR

## Abstract

**Background:**

Q fever, a zoonosis caused by *Coxiella burnetii*, has adverse effects on public health. Ticks are vectors of *C. burnetii* and they contribute to the transmission of the pathogen. A tool for rapid, sensitive, and accurate detection of *C. burnetii* from ticks is important for the prevention of Q fever.

**Methods:**

Ultra-rapid real-time PCR (UR-qPCR) as a chip-based real-time PCR system was developed for the detection of *C. burnetii* from ticks. The UR-qPCR system was established and evaluated for the rapidity, sensitivity, and specificity of *C. burnetii* detection.

**Results:**

*C. burnetii* was detected using UR-qPCR from 5644 larval, nymphal, and adult ticks from 408 pools collected from livestock and epidemiologically linked environments in two provinces, Gangwon and Jeju, in Korea. Ticks from three species were identified; *Haemaphysalis longicornis* accounted for the highest number, present in 333 of 408 pools (81.62%)*,* followed by *Haemaphysalis flava* in 62 pools (15.19%) and *Ixodes nipponensis* in 13 pools (3.19%). The rapidity and sensitivity of PCR detection was demonstrated with the sufficient amplification and detection of approximately 56 copies of *C. burnetii* DNA with only 20 min of PCR amplification. The kappa value for the diagnostic agreement between UR-qPCR and stationary qPCR was in perfect agreement (*κ* = 1). PCR detection and sequencing indicated that *C. burnetii* was present in 5 of the 408 pools (1.23%), in which four pools contained *H. longicornis* and one pool contained *H. flava*. The infection rates of *C. burnetii* in the tick pools collected from Gangwon and Jeju Provinces were 1.70% and 0.58%, respectively. Phylogenetic analysis indicated a close relationship between the detected *C. burnetii* and those originating from goats, humans, and ticks in different countries, such as the USA, France, Germany, and Serbia.

**Conclusions:**

The methods described in this study could be important for the prevention and control of Q fever in the two provinces. The UR-qPCR, with its features of mobility, sensitivity, and rapidity, is helpful for constructing early alert systems in the field for *C. burnetii* in ticks and could help alleviate the transmission of and economic damage due to Q fever.

**Graphic abstract:**

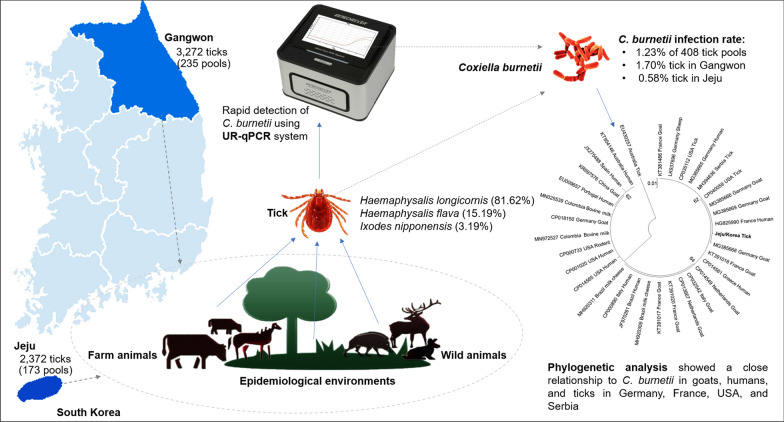

**Supplementary Information:**

The online version contains supplementary material available at 10.1186/s13071-021-04744-z.

## Background

*Coxiella burnetii* is an obligate intracellular bacterium that causes flu-like zoonotic disease [1]. *C. burnetii* infects a variety of vertebrates and is a key threat to veterinary and human health worldwide [2, 3]. The transmission to humans mainly occurs through inhalation of bacteria from contaminated faeces, close contact with livestock, or ingestion of infected animals and animal products [3–8].

More than 40 species of hard and soft ticks are identified as vectors of *C. burnetii* [9, 10]. Body fluids and faeces of ticks contain a large number of infectious *C. burnetii* [11]; as a result, exposure to tick excreta, direct contact with ticks, or tick bite pose potential risks of *C. burnetii* transmission [10, 12]. Therefore, a method for sensitive and accurate detection of *C. burnetii* from ticks is important to prevent infection of humans directly from ticks or indirectly via animals that were hosts of infected ticks [13–15].

Polymerase chain reaction (PCR) and related techniques are widely used as sensitive and specific tools for the detection of *C. burnetii* in ticks, such as conventional PCR [16], restriction fragment length polymorphism (RFLP)-PCR [17, 18], and direct sequencing [19]. The repetitive, transposon-like element, named IS1111, is a specific DNA marker for the sensitive detection of *C. burnetii,* in transposon (Trans)-PCR [5, 20]. PCR-based detection is fast and does not require handling in biosafety level 3 (BL3) cabinets, unlike the isolation of *C. burnetii*. Recently, a chip-based PCR system with optimal thermal transfer was developed to reduce the time needed to perform the assay. The system, named ultra-rapid real-time PCR (UR-qPCR), was demonstrated to be useful for detection of honeybee pathogens [21–23]. The rapidity and mobility of the UR-qPCR could be useful in developing a molecular tool for point-of-care detection of *C. burnetii* from its vectors.

The cases of Q fever diagnosed in humans in Korea have rapidly increased in the subsequent years since the first case was recorded in 2006 [24, 25]. However, there is little information on the tick species that carry *C. burnetii* as well as the regions where *C. burnetii* is present in ticks [26]. Therefore, the present study aimed to detect *C. burnetii* in tick samples collected from different regions of Korea. UR-qPCR, a chip-based real-time PCR, was used for the rapid detection of *C. burnetii* from total nucleic acids extracted from tick samples.

## Methods

### Tick samples

In total, 5644 larval, nymphal, and adult ticks from 408 pools were collected from livestock (cattle and horse) and wild animals (elk, roe deer, raccoon, badger, wild boar, and wild rabbit) from two provinces, Gangwon and Jeju, in Korea between August and November 2019. The 235 pools collected from Gangwon Province were designated as 19M1–19M235, and the 173 samples collected from Jeju Province as 19T1–19T173.

### Identification of tick species

The tick species were identified using a stereo microscope, Discovery.V8 (ZEISS, Oberkochen, Germany). The morphological identification was based on a previously established method [27]. After species identification, samples of the same species collected from the same site were pooled. Each pool contained 1, 10, and 50 ticks of adult, nymph, and larva, respectively. Pools were then stored at −20 °C until the assay for the detection of *C. burnetii* was performed.

### Extraction of total nucleic acid

Total nucleic acids were extracted from tick samples using the Maxwell^®^ RSC Viral Total Nucleic Acid Purification Kit with Maxwell^®^ Instruments (Promega, Madison, WI, USA). One adult tick, 10 nymphs, or 50 larvae were homogenised in a tissue homogeniser using steel beads of diameter 2.381 mm (SNC, Hanam, Korea). The sample was lysed with 330 µl of lysis buffer in a Precellys 24 Tissue Homogeniser (Bertin Instruments, Montigny-le-Bretonneux, France). The homogenate was incubated at 56 °C for 10 min and the following steps were performed using a Maxwell^®^ RSC Instrument, according to the manufacturer’s instructions. Finally, 50 µl of total nucleic acid was obtained from each sample.

### Primers and standard DNA of *C. burnetii*

*Coxiella burnetii* was detected by targeting a 295-bp long DNA fragment in the transposase of the insertion sequence (IS) element IS1111a, using the primers Cox-F (5′-GTCTTAAGGTGGGCTGCGTG-3′) and Cox-R (5′-CCCCGAATCTCATTGATCAGC-3′), and probe Cox-TM (FAM-AGCGAACCATTGGTATCGGACGTT-TAMRA) [28]. DNA from the *C. burnetii* strain 493 (Nine Mile Phase I), preserved in our laboratory, was used as the positive control. The PCR product was cloned in the pGEM^®^-T vector system (Promega, Madison, WI, USA).

### PCR performance

UR-qPCR was performed using a GENECHECKER^®^ UF-300 PCR (Genesystem Co., Ltd., Daejeon, Korea) and 2× Rapi:Spec™ Probe Master mix (Cat. No. 9799100500; Genesystem Co., Ltd.). The reaction mix (10 µl) consisted of 0.4 µl (20 pmol) of each primer, 0.4 µl (2 pmol) of probe, 0.8 µl ddH_2_O, 5 µl PCR premix, and 3 µl of sample total nucleic acid. PCR conditions were set as follows: 95 °C for 30 s, 50 cycles of 95 °C for 5 s, and 60 °C for 10 s. Detection of *C. burnetii* was carried out in two steps, screening and detection. *C. burnetii* was screened from a combination of every five pools of adults, nymphs, or larvae of the same tick species from the same collection site, prepared by combining 10 µl of total nucleic acid from each pool. The individual pools from the PCR-positive combination samples were then reanalysed to identify the exact pool that carried the pathogen.

The performance of the UR-qPCR system was compared to that of the CFX96 Touch Real-time PCR Detection System (Bio-Rad, Hercules, CA, USA) by evaluating the amplification for each of the 408 tick pool nucleic acids using the same primers and probe. The 20 µl reaction mixture consisted of 1 µl (10 pmol) of each primer, 1 µl (5 pmol) of probe, 10 µl of iQ™ Supermix as PCR premix (Bio-Rad), 3 µl of DNA template, and 4 µl of ddH_2_O. PCR conditions were set as follows: 50 °C for 2 min, 95 °C for 10 min, 40 cycles of 95 °C for 15 s, and 60 °C for 30 s. Cohen’s kappa coefficient was used to evaluate the concordance between detection result using UR-qPCR and stationary qPCR. The kappa (*κ*) value is interpreted as follows: 0–0.20 (slight agreement), 0.21–0.40 (fair agreement), 0.41–0.60 (moderate agreement), 0.61–0.80 (substantial agreement), and 0.81–1 (almost perfect agreement) [29, 30].

### Sensitivity and specificity of *C. burnetii* UR-qPCR

To check the sensitivity of UR-qPCR, serial dilutions of *C. burnetii* recombinant DNA template, from 2.8 × 10^8^ to 2.8 × 10^0^ copies, were used for PCR in triplicate to identify the minimum copy number at which the amplification was stable.

To evaluate the specificity of UR-qPCR for *C. burnetii*, DNA from five other tick-borne pathogens (*Anaplasma phagocytophilum*, *Ehrlichia chaffeensis*, *Ehrlichia canis*, *Toxoplasma gondii*, *Borrelia burgdorferi*) were tested with *C. burnetii-*specific primers and probe under the same PCR conditions.

### Sequencing and phylogenetic analysis

The accuracy of UR-qPCR for *C. burnetii* detection was confirmed through sequencing of the PCR products using Cox-F/R primers. The phylogenetic relationship between the detected *C. burnetii* and other reported strains was identified by analysing a 687 bp DNA fragment of the IS1111 transposon gene, which was amplified using the primer pair Trans 1 (5′-TATGTATCCACCGTAGCCAGTC-3′)/Trans 2 (5′-CCCAACAACACCTCCTTATTC-3′) [31, 32]. The sequences were compared to the available *C. burnetii* sequences in NCBI using the Basic Local Alignment Search Tool (BLAST). Consensus sequences were aligned using the Clustal X2 program [33], overhanging ends were trimmed using BioEdit 7.2 [34], and a maximum likelihood phylogenetic tree was created using MEGA7 [35], the Kimura two-parameter model, gamma distribution, and bootstrapping 1000 times.

## Results

### Sensitivity and specificity of *C. burnetii*-specific UR-qPCR

**Fig. 1:**
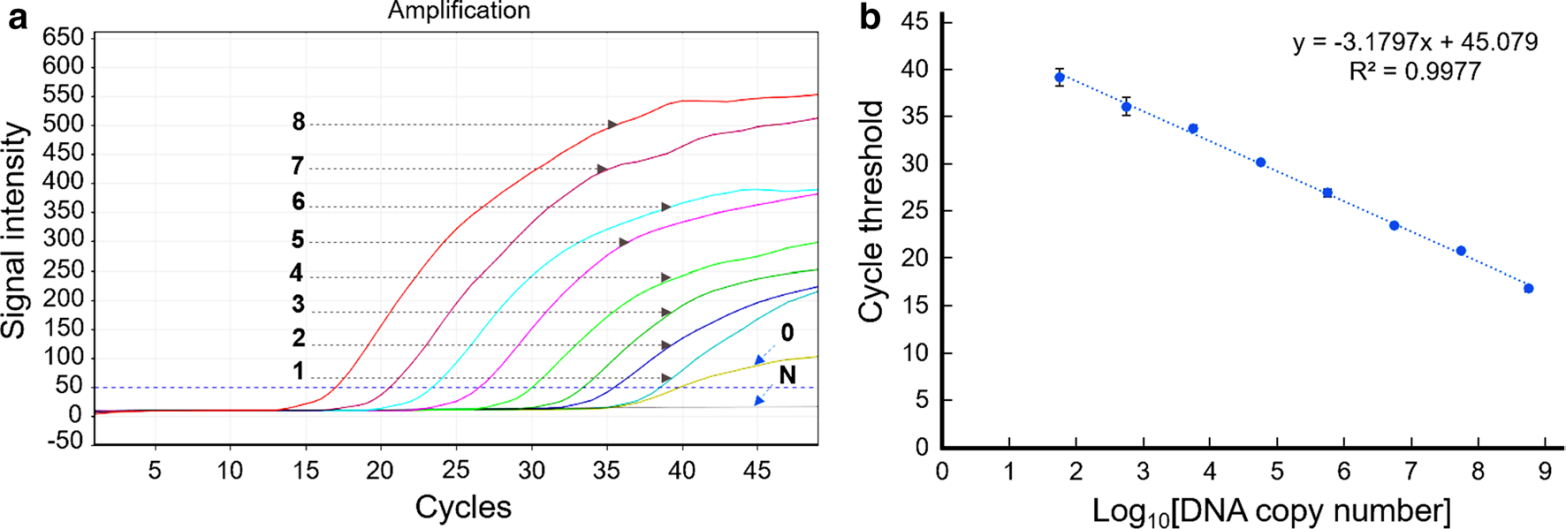
Sensitivity of UR-qPCR for detection of *Coxiella burnetii*. **a** The amplification curves indicate the amplification of *C. burnetii* recombinant DNA, serially diluted from 5.6 × 10^8^ to 5.6 × 10^0^ copies, denoted by numbers 8 to 0, respectively. “N” is negative control without DNA template. **b** Standard linear regression graph representing the relationship between initial DNA copy number and Ct value, averaged from PCR reactions performed in triplicate

UR-qPCR could stably detect *C. burnetii* DNA at 5.6 × 10^1^ copies with a cycle threshold (Ct) of less than 40 (Fig. 1a). The linear regression representing the relationship between initial DNA copy number and Ct from triplicate PCR reactions was determined by *y* = −3.1797*x* + 45.079; *R*^2^ = 0.9977, where* y* and *x* are the Ct value and log_10_ DNA copy number, respectively (Fig. 1b). The amplification efficiency calculated from the slope of the standard curve (*E* = 10^(−1/slope)^ −1) was 106.30%.

**Fig. 2:**
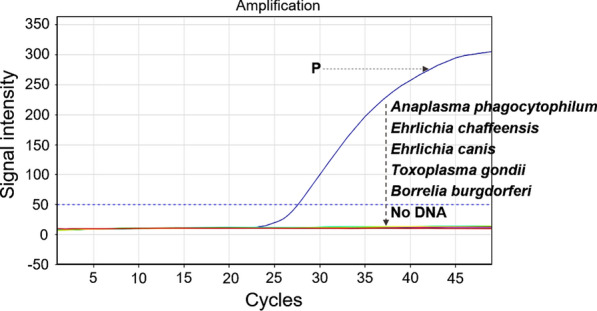
Specificity of UR-qPCR for detection of *Coxiella burnetii*. The specificity of UR-qPCR for the detection of *C. burnetii* was confirmed by the lack of amplification from the DNA templates of five other tick-borne pathogens (*Anaplasma phagocytophilum*, *Ehrlichia chaffeensis*, *Ehrlichia canis*, *Toxoplasma gondii*, *Borrelia burgdorferi*) and in the negative control, which lacked the DNA template. “P” is the positive control containing 10^5^ copies of *C. burnetii* recombinant DNA

The specificity of the UR-qPCR system for *C. burnetii* detection was confirmed by the lack of cross detection of any of the five other tick-borne pathogens (Fig. 2). Therefore, the UR-qPCR system could be a potential molecular tool for the rapid, sensitive, and specific detection of *C. burnetii* from ticks and for the diagnosis of Q fever.

### Infection rate of *C. burnetii* in tick samples

Ticks from 408 pools were evaluated; the major species was *Haemaphysalis longicornis* (Asian longhorned tick), present in 333 pools (81.62%), followed by *H. flava*, in 62 pools (15.19%), and *Ixodes nipponensis* (Japanese hard tick), in 13 pools (3.19%). *I. nipponensis* was observed only in the pools from Gangwon Province. Five of the 408 tick pools (1.23%) carried *C. burnetii,* in which four pools originated from wild animals and epidemiologically linked environments in Gangwon Province (19M22, 19M42, 19M73, and 19M88) and one pool (19T112) was collected from the cattle in Jeju Province (Table 1).

**Table 1:** *C. burnetii *infection rate in ticks collected from Jeju and Gangwon province in 2019

Province	Species	Stage	No. of ticks (no. of tested pools)	No. of positive pools (%)	
				UR-qPCR	qPCR
Gangwon	*H. longicornis*	Larva	2764 (53)	1 (1.89)	1 (1.89)
		Nymph	50 (14)	0	0
		Adult (male)	38 (15)	0	0
		Adult (female)	240 (122)	2 (1.64)	2 (1.64)
	*H. flava*	Larva	0	0	0
		Nymph	93 (7)	0	0
		Adult (male)	28 (5)	1 (20)	1 (20)
		Adult (female)	10 (6)	0	0
	*I. nipponensis*	Larva	0	0	0
		Nymph	16 (2)	0	0
		Adult (male)	3 (1)	0	0
		Adult (female)	30 (10)	0	0
Jeju	*H. longicornis*	Larva	1470 (23)	1 (4.35)	1 (4.35)
		Nymph	158 (13)	0	0
		Adult (male)	99 (33)	0	0
		Adult (female)	208 (60)	0	0
	*H. flava*	Larva	0	0	0
		Nymph	368 (18)	0	0
		Adult (male)	30 (8)	0	0
		Adult (female)	39 (18)	0	0
Total			5644 (408)	5 (1.23)	5 (1.23)

The rate of *C. burnetii* infection was 1.20% (4/333 pools), 1.61% (1/62 pools), and 0% (0/13 pools) in *H. longicornis, H. flava*, and *I. nipponensis*, respectively. *C. burnetii* infection was detected in the larval and adult stages of *H. longicornis* at 2.63% (2/76 pools) and 0.87% (2/230 pools), respectively, however, only in the adult stage of *H. flava* at 2.70% (1/37 pools) (Table 2).

**Table 2:** Detection rate of *Coxiella burnetii* from tick species

Species	Stage	No. of ticks (no. of tested pools)	No. of positive pools (%)	
			UR-qPCR	qPCR
*H. longicornis*	Larva	4234 (76)	2 (2.63)	2 (2.63)
	Nymph	208 (27)	0	0
	Adult (male)	137 (48)	0	0
	Adult (female)	448 (182)	2 (1.10)	2 (1.10)
*H. flava*	Larva	0	0	0
	Nymph	461 (25)	0	0
	Adult (male)	58 (13)	1 (7.69)	1 (7.69)
	Adult (female)	49 (24)	0	0
*I. nipponensis*	Larva	0	0	0
	Nymph	16 (2)	0	0
	Adult (male)	3 (1)	0	0
	Adult (female)	30 (10)	0	0
Total		5644 (408)	5 (1.23)	5 (1.23)

**Fig. 3:**
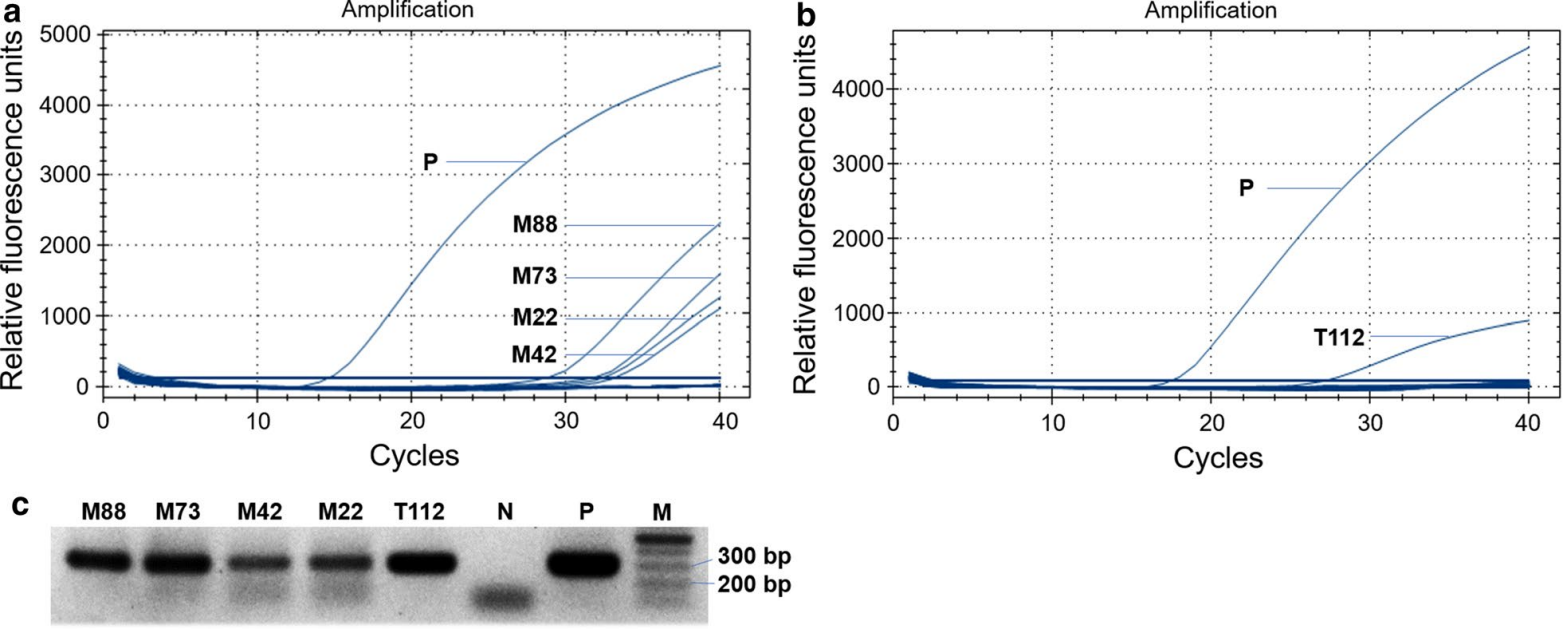
Confirmation of *Coxiella burnetii* detection from tick samples. Detection curves indicate that the same five tick samples are positive for *C. burnetii* when tested using the CFX96 Touch Real-time PCR System (Bio-Rad). **a** Four samples (19M88, 19M73, 19M42, 19M22) from Gangwon Province. **b** One sample (19T112) from Jeju Province. “P” is the positive control using *C. burnetii* recombinant DNA. **c** Electrophoresis of PCR amplicons (295 bp long) from the five samples (19M88, 19M73, 19M42, 19M22, 19T112) positive for *C. burnetii*. “P” and “N” are the positive control and the negative control, without DNA template, respectively, and “M” is the 100 bp DNA marker

The accuracy of *C. burnetii* detection in UR-qPCR was consistent with that of conventional real-time PCR (qPCR; CFX96 Touch Real-time PCR; Bio-Rad). The same five tick pools (19M22, 19M42, 19M73, 19M88, and 19T112) were positive (Table 1; Fig. 3). The Cohen’s kappa coefficient calculated from the results of UR-qPCR and qPCR was *κ* = 1, indicating a near perfect agreement (0.81–1.00).

### Sequencing and phylogenetic analysis

Sequencing the 295 bp amplicons confirmed the accuracy of detection. The amplicon from the 19M22, 19M42, 19M73, and 19T112 pools showed 99% DNA sequence identity with the sequence from *C. burnetii* strain RSA493 (NCBI accession number CP040059); while the sequence from the 19M88 pool showed 100% identity to the sequence from *C. burnetii* strain BTM90C (NCBI accession number MN025541) (Additional file 1).

**Fig. 4:**
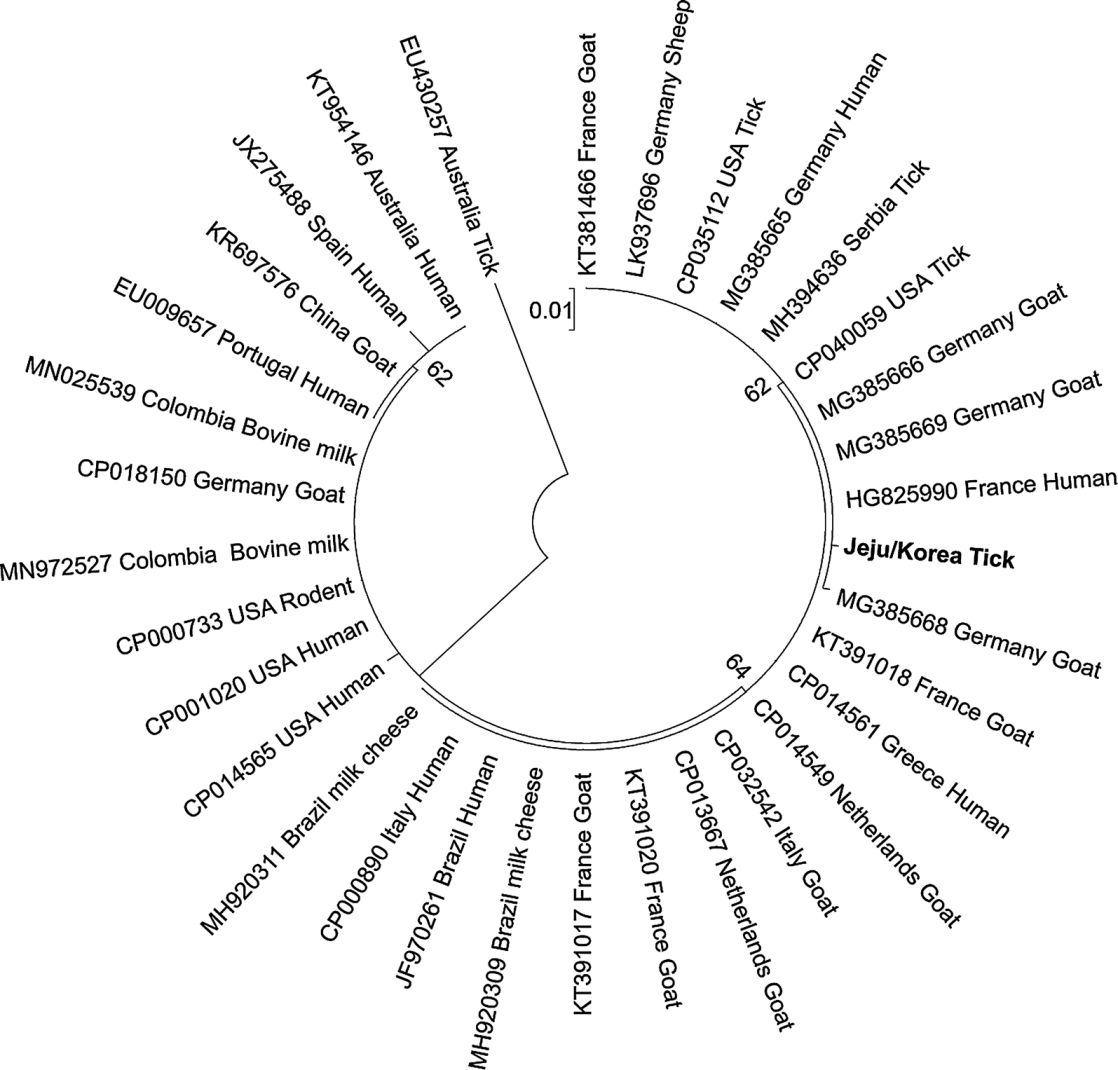
Maximum likelihood phylogenetic tree based on the partial IS1111 nucleotide sequences (643 bp) of *Coxiella burnetii* from the Republic of Korea and other countries. The sequences were aligned using Clustal X2, edited using BioEdit, and maximum likelihood tree generated using MEGA7 software, bootstrapping 1000 times

The analysis of the 687 bp fragment showed homologies ranging from 97.43 to 99.85% to the sequences of *C. burnetii* in NCBI. The detected *C. burnetii* was closely related to and clustered with strains originated from ticks, goats, and humans in the USA, France, Germany, and Serbia, on the phylogenetic tree (Fig. 4).

## Discussion

A chip-based PCR system, UR-qPCR, was introduced in this study for the rapid detection of the tick-borne Q fever pathogen. The UR-qPCR system is rapid, requiring approximately 20 min for 50 cycles, compared to the approximately 1 h and 30 min required for the other PCR systems compared in this study. The rapidity and mobility of the UR-qPCR system could be useful for development of a molecular tool for on-site surveillance of *C. burnetii* in ticks. In addition, targeting IS1111, a multi-copy element in *C. burnetii* [28, 36], increases the sensitivity of the UR-qPCR system. It is believed that the use of IS1111 is limited by its presence in *Coxiella*-like bacteria (CLB) and *Rickettsiella* spp. [37, 38]; however, we showed that the CLB in ticks does not interfere with the accuracy of *C. burnetii* detection using sequencing, and the comparison of the *C. burnetii*-specific primers used in this study to genomes of *Rickettsiella* spp. showed that there was no amplification of the primers on *Rickettsiella* genome with the amplicon size that could interfere with the accurate detection of *C. burntetii* (Additional file 1: Fig. S1).

The diagnosis of Q fever relies mainly on serology [39]. However, serological diagnosis can be unreliable due to the cross-reaction with *Bartonella* spp., *Ehrlichia* spp., and *Rickettsia* spp. [40]. PCR is a useful detection tool for improving the accuracy of a diagnosis [20, 41]; PCR detection of *C. burnetii* in blood was effective in diagnosing Q fever with a sensitivity of approximately 81% compared to indirect immunofluorescence assay (IFA) [42]. Therefore, the sensitivity and rapidity of the *C. burnetii*-specific UR-qPCR system could be beneficial for on-site conformational diagnosis of Q fever, for the prompt control of milk, blood, or serum samples.

Detection of *C. burnetii* using loop-mediated isothermal amplification (LAMP) assay [43, 44] is rapid and comparable to real-time PCR. The positive detection is based on a change of colour in the reaction mix after 30 min incubation. However, the sensitivity of the colorimetric LAMP assay is only 93.75% compared to that of real-time PCR. Therefore, the UR-qPCR proposed in this study has the advantages of being rapid, with less than 20 min reaction time, and sensitive, at 100% sensitivity compared to other real-time PCR systems. The UR-qPCR and the crude DNA preparation [44] together will take less than 30 min for the detection of *C. burnetii* on-site.

There is only one previous report of a *H. longicornis* sample, from Cheongju city in Chungcheongbuk Province in Korea, harbouring *C. burnetii* [45]. However, this study revealed that two (*H. longicornis* and *H. flava*) of the three prevalent tick species (*H. longicornis*, *H. flava,* and *I. nipponensis*) [46] in two provinces (Gangwon and Jeju) harbour the Q fever pathogen. *C. burnetii* was detected in tick samples from wild animals, livestock, and grasslands with one and four pools in Jeju and Gangwon Provinces, respectively, although no case of Q fever in humans has been recorded in Jeju Province [24]. These provinces have a high risk of *C. burnetii* transmission through the ticks harbouring the pathogen and parasitizing cattle.

## Conclusions

A rapid real-time PCR assay was developed for the detection of the Q fever pathogen, *C. burnetii*, in tick species collected from two provinces in Korea. The rapidity and accuracy of this PCR system was evaluated. The automated nucleic acid isolation system used in this study minimized the exposure to living bacteria in ticks, which could pose a risk of *C. burnetii* infection during the sample DNA preparation*. C. burnetii* was detected in two tick species (*H. longicornis* and *H. flava*), which are parasites in wild animals and from the grasslands in Gangwon Province, and in *H. longicornis* from cattle in Jeju Province. This information is important for the prevention of Q fever, particularly in Jeju Province, where no case of infection in humans has been recorded.

## Supplementary Information

**Additional file 1: Table S1.** *Coxiella burnetii* sequences detected from tick samples. **Figure S1.** Comparison of *Coxiella burnetii-*specific primers to genome of *Rickettsiella* species. The comparison was performed by aligning the forward and reverse primers of *C. burnetii* to the genome of *R. viridis* (A), *R. grylli* (B), and *R. isopodorum* (C). Numbers indicate the positions on *Rickettsiella* genomes that had the highest similarity to the primer sequences. The distance between the forward and reverse primer positions are indicates as base pairs.

## Data Availability

All data generated or analysed during this study are included in this published article and Additional file 1: Table S1 and Fig. S1.

## Abbreviations

IS1111: Insertion sequence 1111
CLB: *Coxiella-*Like bacteria
KDCA: Korea Disease Control and Prevention Agency
UR-qPCR: Ultra-rapid real-time polymerase chain reaction
RFLP: Restriction fragment length polymorphism
Ct: Cycle threshold
LAMP: Loop-mediated isothermal amplification
