# Supplementary material for: Real-time PCR biochip for on-site detection of Coxiella burnetii in ticks
Source: Parasit Vectors. 2021 May 6;14:239. doi: 10.1186/s13071-021-04744-z (PMC8101159; doi:10.1186/s13071-021-04744-z)
Supplement: Supplementary file 1 — Additional file 1: Table S1. Coxiella burnetii sequences detected from tick samples. Figure S1. Comparison of Coxiella burnetii-specific primers to genome of Rickettsiella species. The comparison was performed by aligning the forward and reverse primers of C. burnetii to the genome of R. viridis (A), R. grylli (B), and R. isopodorum (C). Numbers indicate the positions on Rickettsiella genomes that had the highest similarity to the primer sequences. The distance between the forward and reverse primer positions are indicates as base pairs. [file 13071_2021_4744_MOESM1_ESM.docx]

**Additional file 1**

**Table S1.** *Coxiella burnetii* sequences detected from tick samples

| **Sample name** | **Sequence (5′-3′)** | **NCBI reference** |
| --- | --- | --- |
| 19M22, 19M42, 19M73 | GTCTTAAGGTGGGCTGCGTGGTGATGGAAGCGTGTGGAGGAGCGAACCATTGGTATCGGACGTTTATGGGGATGGGTATCCCAACGCAGTTGATCAGTCCGCAGCACGTCAAACCGTATGTCAAAAGTAACAAGAATGATCGTAACGATGCGCAGGCGATAGCTGAAGCGGCTTCCCGCGCCTCGATGCGGTTTGTGCAGGGTAAAACGGTGGAACAACAAGACGTTCATGCGCTGATAAAGATACGCGATCGTTTAGTCAAAAGCCGCACGGCGCTGATCAATGAGATTCGGGG | 99% identity with the sequence from *C. burnetii* strain RSA439, NCBI accession number CP040059 |
| 19M88 | GTCTTAAGGTGGGCTGCGTGGTGATGGAAGCGTGTGGAGGAGCGAACCATTGGTATCGGACGTTTATGGGGATGGGTATCCCAACGCAGTTGATCAGTCCGCAGCACGTCAAACCGTATGTCAAAAGTAACAAGAATGATCGTAACGATGCGCAGGCGATAGCTGAAGCGGCTTCCCGCGCCTCGATGCGGTTTGTGCGGGGTAAAACGGTGGAACAACAAGACGTTCAAGCGCTGTTAAAGATACGCGATCGTTTAGTCAAAAGCCGCACGGCGCTGATCAATGAGATTCGGGG | 100% identity with the sequence from *C. burnetii* strain BTM90C, NCBI accession number MN025541 |
| 19T112 | GTCTTAAGGTGGGCTGCGTGGTGATGGAAGCGTGTGGAGGAGCGAACCATTGGTATCGGACGTTTATGGGGATGGGTATCCCAACGCAGTTGATCAGTCCGCAGCACGTCAAACCGTATGTCAAAAGTAACAAGAATGATCGTAACGATGCGCAGACGATAGCTGAAGCGGCTTCCCGCGCCTCGATGCGGTTTGTGCAGGGTAAAACGGTGGAACAACAAGACGTTCAAGCGCTGTTAAAGATACGCGATCGTTTAGTCAAAAGCCGCACGGCGCTGATCAATGAGATTCGGGG | 99.66% identity with the sequence from *C. burnetii* strain RSA439, NCBI accession number CP040059 |
| 19T112* | TATGTATCCACCGTAGCCAGTCTTAAGGTGGGCTGCGTGGTGATGGAAGCGTGTGGAGGAGCGAACCATTGGTATCGGACGTTTATGGGGATGGGTATCCCAACGCAGTTGATCAGTCCGCAGCACGTCAAACCGTATGTCAAAAGTAACAAGAATGATCGTAACGATGCGCAGACGATAGCTGAAGCGGCTTCCCGCGCCTCGATGCGGTTTGTGCAGGGTAAAACGGTGGAACAACAAGACGTTCAAGCGCTGTTAAAGATACGCGATCGTTTAGTCAAAAGCCGCACGGCGCTGATCAATGAGATTCGGGGGTTGTTGCAAGAATACGGACTCACGATGGCGCGTGGTGCCAAGCGATTTTATGAAGAGCTCCCGTTGATTTTAGCGAGCGAAGCGGTGGGATTAACACCGCGGATGAAACGGGTGTTGAATTGTTTGTATACCGAATTGTTGAACCGGGACGAAGCGATTGGTGATTACGAGGAGGAATTAAAAGCGGTGGCAAAAGCCAATGAGGATTGTCAACGGGTACAGAGCATCCCGGGGGTGGGTTATTTAACGGCGCTCTCGGTTTATGCGAGCGTGGGTGACATTCATCAATTTCATCGTTCCCGGCAGTTGTCGGCGTTTATTGGGTTGGTCCCTCGACAACATTCGAGTGGGAATAAGGAGGTGTTGTTGGG | 99.85% identity with the sequence from *C. burnetii* strain RSA439, NCBI accession number CP040059 |

*Phylogenetic analysis sequence and sequences confirmation of UR-qPCR results


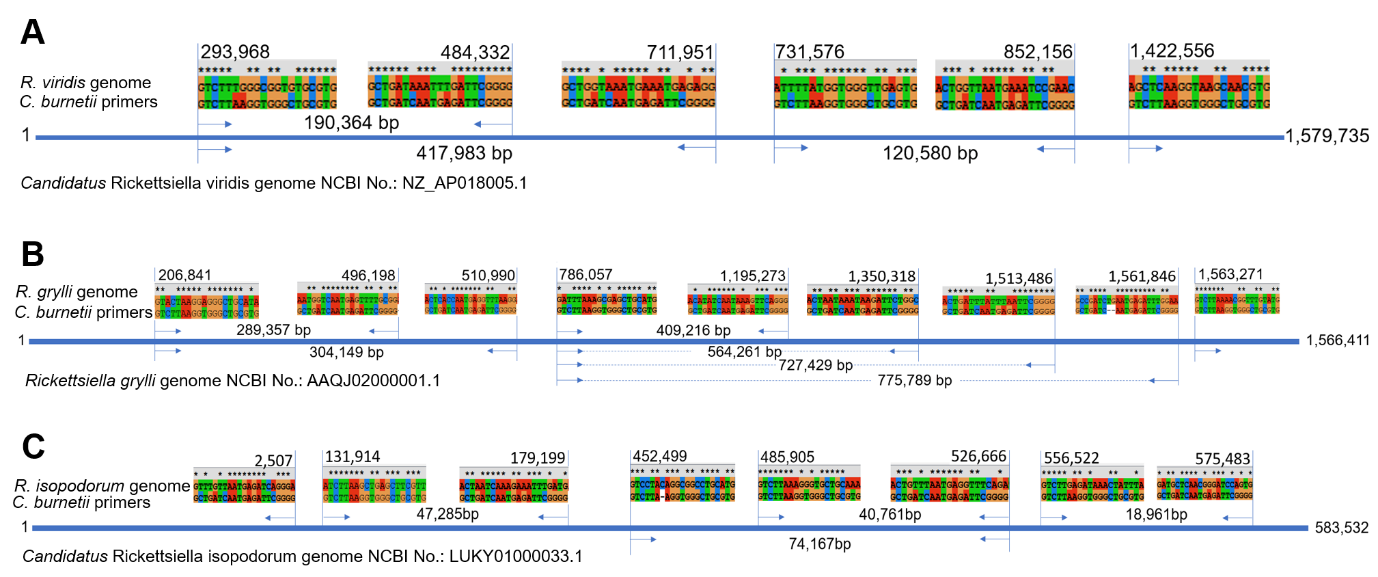


Fig. S1. Comparison of *Coxiella burnetii-*specific primers to genome of *Rickettsiella* species. The comparison was performed by aligning the forward and reverse primers of *C. burnetii* to the genome of *R. viridis* (A), *R. grylli* (B), and *R. isopodorum* (C). Numbers indicate the positions on *Rickettsiella* genomes that had the highest similarity to the primer sequences. The distance between the forward and reverse primer positions are indicates as base pairs.
